# Evaluation of the Humoral Immune Response to Human Leukocyte Antigens in Brazilian Renal Transplant Candidates

**DOI:** 10.1371/journal.pone.0100270

**Published:** 2014-06-13

**Authors:** Patricia Keiko Saito, Roger Haruki Yamakawa, Erica Pereira Aparecida, Waldir Verissimo da Silva Júnior, Sueli Donizete Borelli

**Affiliations:** 1 Department of Basic Health Sciences, Universidade Estadual de Maringá, Maringá, Paraná, Brazil; 2 Histogene Laboratory of Histocompatibility and Genetics, Maringá, Paraná, Brazil; 3 Department of Statistics, Universidade Estadual de Maringá, Maringá, Paraná, Brazil; University of Sao Paulo Medical School, Brazil

## Abstract

Pre-transplant sensitization to human leukocyte antigens (HLA) is a risk factor for graft failure. Studies of the immunological profile related to anti-HLA antibodies in Brazilian renal transplant candidates are few. In this study, we evaluated the humoral immune response to HLA antigens in 269 renal transplant candidates, in Paraná State, Brazil. The HLA typing was performed by the polymerase chain reaction sequence-specific oligonucleotide method (PCR-SSO) combined with Luminex technology, using an SSO-LABType commercial kit (One Lambda, Inc., Canoga Park, CA, USA). The percentages of panel-reactive antibodies (PRA) and the specificity of anti-HLA antibodies were determined using the LS1PRA and LS2PRA commercial kits (One Lambda, Inc.). The PRA-positive group consisted of 182 (67.7%) patients, and the PRA-negative group of 87 (32.3%) patients. The two groups differed significantly only with respect to gender. Females were the most sensitized. Among the 182 patients with PRA- positive, 62 (34.1%) were positive for class I and negative for class II, 39 (21.4%) were negative for class I and positive for class II, and 81 (44.5%) were positive for both classes I and II. The HLA-A*02, A*24, A*01, B*44, B*35, B*15, DRB1*11, DRB1*04 and DRB1*03 allele groups were the most frequent. The specificities of anti-HLA antibodies were more frequent: A34, B57, Cw15, Cw16, DR51, DQ8 and DP14. This study documented the profile of anti-HLA antibodies in patients with chronic renal failure who were on waiting lists for an organ in Paraná, and found high sensitization to HLA antigens in the samples.

## Introduction

The importance of anti-human leukocyte antigen (HLA) antibodies in organ transplantation has been known since the 1960s [1]. Many studies have shown that individuals who undergo a hyperacute rejection of an organ, or episodes of acute rejection, whether of the first or a subsequent graft, may contain anti-HLA antibodies in their serum [1]–[3]. These antibodies can be formed as a result of pregnancies [4]–[6], blood transfusions [6], [7], and previous transplants [6], [8].

Detection of anti-HLA antibodies is an important tool for successful transplantation [2], [9], since pre-transplant sensitization to HLA antigens is a risk factor for graft failure [10], [11]. Patients with high percentages of panel-reactive antibodies (PRA) have a higher probability of rejection [10]–[12].

Studies on the immunological profile of anti-HLA class I (A, B and Cw) and class II (DR, DQ and DP) antibodies in Brazilian renal transplant candidates are few, in particular in the state of Paraná, Brazil. This study evaluated the immune response to HLA antigens in patients with chronic renal failure, renal transplant candidates, in northern and northwestern Paraná, southern Brazil.

## Materials and Methods

### Patients

The study was conducted with 269 patients with chronic renal failure, renal transplant candidates, from northern and northwestern Paraná in southern Brazil. Only patients with updated records (active patients/potential recipients) were included. Data regarding demographic characteristics, and potential risk factors for the development of anti-HLA antibodies (transfusions, pregnancies and previous transplants) were obtained from the records of dialysis clinics.

According to the results for the percentage of panel-reactive antibodies (PRA), the patients were divided into two groups, PRA-negative (PRA = 0) and PRA-positive (PRA>0). Subsequently, the PRA-positive patients were divided into 2 groups according to the level of PRA class I and class II, considered separately. The first group included patients with PRA from 1% to 50%, and the second group included patients with PRA between 51% and 100%.

This study was approved by the Ethics Committee of the Universidade Estadual de Maringá, Paraná, Brazil (protocol no. 333/2011). All procedures followed Resolution 196/1996 of the Brazilian Health Council, which rules on research on humans in Brazil. All procedures were explained to each subject, and written informed consent was obtained from each subject.

### DNA Extraction and HLA Class I and II Typing

To perform the HLA typing, 5 mL of peripheral blood was collected by venipuncture into vacuum tubes (Vacutainer, Becton and Dickson, Oxford, England) containing ethylene diamine tetraacetic acid (EDTA) as an anticoagulant. Genomic DNA extraction was performed by the separation column method, using a Biopur commercial kit for DNA extraction (Biometrix, Curitiba, Paraná, Brazil), following the manufacturer’s protocol. After the concentration of the DNA obtained was adjusted by the optical-density method, the polymerase chain reaction sequence-specific oligonucleotide method (PCR-SSO) combined with Luminex technology was carried out using SSO-LABType HLA class I (HLA-A, -B) and class II (HLA-DRB1) commercial kits (One Lambda, Inc., Canoga Park, CA, USA), which provide low-to-medium resolution typing, following the manufacturer’s protocol. In this method, genomic DNA was amplified using a biotinylated sequencing primer locus-specific for HLA class I (HLA-A and -B) and class II (HLA-DRB1) in a GeneAmp PCR System 9700 thermocycler (Applied Biosystems, Foster City, CA, USA). Subsequently, hybridization was performed with complementary DNA probes conjugated to microspheres (beads) labeled with different fluorochromes to identify complementary sequences of the amplified DNA. After hybridization, the samples were read by means of the flow cytometry platform LABScan^TM^100 (One Lambda, Inc.), followed by analysis by HLA Fusion software version 2.0 (One Lambda, Inc.).

### Determination of Percentages of Panel-Reactive Antibodies (PRA) and HLA-specific Antibodies

The percentages of reactivity of panel-reactive antibodies (PRA) and HLA-specific antibodies were determined in sera from the patients, using the LS1PRA and LS2PRA commercial kits (One Lambda, Inc.) combined with the Luminex technology, following the manufacturer’s protocol. In this method, percentages of PRA and HLA-specific antibodies were identified using a panel of color-coded beads coated with purified HLA antigens. The test serum was first incubated with these beads. Any HLA antibodies present in the test serum were bound to the antigens and then were labeled with R-Phycoerythrin (PE)-conjugated Goat anti-human IgG. The fluorescent emission of PE from each bead was detected using the flow cytometry platform LABScanTM100 (One Lambda, Inc.), followed by analysis in HLA Fusion version 2.0 software (One Lambda, Inc.) using the fluorescence analysis minimum recommended by the manufacturer (median fluorescence intensity equal to or greater than 500).

### Statistical Analyses

All statistical analyses were performed with Statistica 7 software. Allele and phenotype frequencies were calculated by direct counting. Fisher’s exact test and Student’s T test were used to compare the demographic characteristics and potential risk factors for the development of anti-HLA antibodies among the study groups. The significance level of the statistical test was 5% (*P*<0.05).

## Results

The demographic characteristics and potential risk factors for the development of anti-HLA antibodies, according to the PRA results, are shown in Table 1.

**Table 1:** Demographic characteristics and potential risk factors for the development of anti-HLA antibodies, according to the PRA results.

	PRA - Positive	PRA - Negative	*P* - value
	(n = 182)	(n = 87)	
Mean age (years)	52.0±13.2	52.4±12.0	
Gender			
Males	106 (58.2%)	66 (75.9%)	
Females	76 (41.8%)	21 (24.1%)	0.0064
Ethnic group			
White (Caucasian)	104 (57.1%)	52 (59.7%)	
Brown (Mestizo)	54 (29.7%)	16 (18.4%)	
Black	20 (11.0%)	14 (16.1%)	
Yellow (Oriental)	4 (2.2%)	5 (5.8%)	
Potential risk factors			
Mean dialysis duration (years)	7.0±3.8	6.3±3.4	
Any transfusions (no. of patients)	110 (60.4%)	50 (57.5%)	
Number of blood transfusions (units)	3.14±7.05	2.16±3.85	
Any pregnancies (no. of females)	62 (81.6%)	15 (71.4%)	
Previous transplantation history (no. of patients)	16 (8.8%)	4 (4.6%)	

The PRA-positive group consisted of 182 (67.7%) patients, and the PRA-negative group of 87 (32.3%) patients. Only gender showed a significant difference between the two groups. The females were the most sensitized.

The frequencies of the HLA-A, -B and -DRB1 allele groups of the patients are shown in Table 2. The HLA-A*02, A*24 and A*01 allele groups were the most common locus for A. In the B locus, the most common allele groups were HLA-B*44, B*35, and B*15; and in the DRB1 locus, the HLA-DRB1*11, DRB1*04 and DRB1*13 allele groups were the most common.

**Table 2:** HLA-A, -B and -DRB1 alleles and phenotype frequencies of the total patient group.

HLA-A*	Allele frequencies	Phenotype frequencies	HLA-B*	Allele frequencies	Phenotype frequencies	HLA-DRB1*	Allele frequencies	Phenotype frequencies
*01*	0.1041	0.2082	*07*	0.0613	0.1227	*01*	0.0855	0.1710
*02*	0.2416	0.4833	*08*	0.0558	0.1115	*03*	0.1097	0.2193
*03*	0.0892	0.1784	*13*	0.0186	0.0372	*04*	0.1338	0.2677
*11*	0.0651	0.1301	*14*	0.0260	0.0520	*07*	0.1115	0.2230
*23*	0.0446	0.0892	*15*	0.0874	0.1747	*08*	0.0558	0.1115
*24*	0.1152	0.2305	*18*	0.0390	0.0781	*09*	0.0204	0.0409
*25*	0.0316	0.0632	*27*	0.0242	0.0483	*10*	0.0260	0.0520
*26*	0.0372	0.0743	*35*	0.1041	0.2082	*11*	0.1357	0.2714
*29*	0.0242	0.0483	*37*	0.0112	0.0223	*12*	0.0093	0.0186
*30*	0.0520	0.1041	*38*	0.0279	0.0558	*13*	0.1301	0.2602
*31*	0.0335	0.0669	*39*	0.0409	0.0818	*14*	0.0446	0.0892
*32*	0.0279	0.0558	*40*	0.0651	0.1301	*15*	0.1078	0.2156
*33*	0.,0279	0.0558	*41*	0.0186	0.0372	*16*	0.0297	0.0595
*34*	0.0093	0.0186	*42*	0.0242	0.0483			
*36*	0.0074	0.0149	*44*	0.1059	0.2119			
*66*	0.0112	0.0223	*45*	0.0223	0.0446			
*68*	0.0576	0.1152	*47*	0.0019	0.0037			
*69*	0.0019	0.0037	*48*	0.0093	0.0186			
*74*	0.0149	0.0297	*49*	0.0316	0.0632			
*80*	0.0037	0.0074	*50*	0.0335	0.0669			
			*51*	0.0836	0.1673			
			*52*	0.0204	0.0409			
			*53*	0.0242	0.0483			
			*54*	0.0056	0.0112			
			*55*	0.0112	0.0223			
			*56*	0.0019	0.0037			
			*57*	0.0260	0.0520			
			*58*	0.0149	0.0297			
			*81*	0.0019	0.0037			
			*82*	0.0019	0.0037			

The distribution of anti-HLA antibodies according to the percentage of PRA is shown in Table 3.

**Table 3:** Distribution of anti-HLA antibodies according to the percentage of PRA.

	Group 1 PRA = 1%–50%	Group 2 PRA = 51%–100%
**Anti-Class I** (N = 143)		
Anti-locus A	23	4
Anti-locus B	42	0
Anti-locus C	1	0
Anti-locus A+B	26	29
Anti-locus A+Cw	0	0
Anti-locus B+Cw	2	0
Anti-locus A+B+Cw	3	13
**Anti-class II**(N = 120)		
Anti-locus DR	21	4
Anti-locus DQ	10	1
Anti-locus DP	0	0
Anti-locus DR+DQ	36	38
Anti-locus DR+DP	0	0
Anti-locus DQ+DP	0	0
Anti-locus DR+DQ+DP	2	8

Among the 182 patients with positive PRA, 62 (34.1%) were positive for class I and negative for class II, 39 (21.4%) were negative for class I and positive for class II, and 81 (44.5%) were positive for both classes I and II. According to the percentages of PRA class I, 97 (67.8%) patients were in group 1 and 46 (32.2%) were in group 2. For class II, 69 (57.5%) patients were in group 1 and 51 (42.5%) were in group 2.

Furthermore, according to the percentages of PRA greater or equal to 80, 28 patients were positive for class I, 27 patients for class II, and 11 patients for classes I and II.

The overall incidences of anti-HLA-A, -B, -Cw, -DR, -DQ and -DP antibodies in the group of patients with positive PRA are shown in Tables 4 and 5. The specificities of anti-HLA antibodies were most frequent: A34, B57, Cw15, Cw16, DR51, DQ8 and DP14.

**Table 4:** Anti-HLA class I antibodies in patients with a positive PRA.

anti-HLA-A	frequency	anti-HLA-B	frequency	anti-HLA-Cw	frequency
anti-A1	0.1758	anti-B7	0.1484	anti-Cw1	0.0055
anti-A2	0.2143	anti-B8	0.1374	anti-Cw4	0.0220
anti-A3	0.0714	anti-B13	0.0989	anti-Cw6	0.0110
anti-A11	0.0989	anti-B18	0.1044	anti-Cw7	0.0055
anti-A23	0.1978	anti-B27	0.1703	anti-Cw9	0.0055
anti-A24	0.2308	anti-B35	0.1538	anti-Cw12	0.0110
anti-A25	0.1319	anti-B37	0.0330	anti-Cw14	0.0110
anti-A26	0.0879	anti-B38	0.0495	anti-Cw15	0.0330
anti-A29	0.0769	anti-B39	0.1044	anti-Cw16	0.0330
anti-A30	0.0769	anti-B41	0.0440	anti-Cw18	0.0055
anti-A31	0.1044	anti-B42	0.1374		
anti-A32	0.1319	anti-B44	0.0934		
anti-A33	0.1264	anti-B45	0.0934		
anti-A34	0.2473	anti-B46	0.0055		
anti-A36	0.1154	anti-B47	0.0385		
anti-A66	0.1429	anti-B48	0.1319		
anti-A68	0.2253	ant-B49	0.1154		
anti-A69	0.1868	anti-B50	0.1209		
anti-A74	0.0385	anti-B51	0.1209		
anti-A80	0.0824	anti-B52	0.1264		
		anti-B53	0.1154		
		anti-B54	0.1044		
		anti-B55	0.1044		
		anti-B56	0.1484		
		anti-B57	0.2088		
		anti-B58	0.1813		
		anti-B59	0.0604		
		anti-B60	0.1593		
		anti-B61	0.1374		
		anti-B62	0.0934		
		anti-B63	0.1319		
		anti-B64	0.0769		
		anti-B65	0.1044		
		anti-B67	0.1538		
		anti-B71	0.0659		
		anti-B72	0.0659		
		anti-B73	0.0275		
		anti-B75	0.0604		
		anti-B76	0.0659		
		anti-B78	0.0989		
		anti-B81	0.1374		
		anti-B82	0.0769		

**Table 5:** Anti-HLA class II antibodies in patients with a positive PRA.

anti-HLA-DR	frequency	anti-HLA-DQ	frequency	anti-HLA-DP	frequency
anti-DR1	0.2308	anti-DQ2	0.0934	anti-DP1	0.0055
anti-DR4	0.2198	anti-DQ4	0.0769	anti-DP2	0.0055
anti-DR7	0.2143	anti-DQ5	0.0714	anti-DP3	0.0110
anti-DR8	0.2198	anti-DQ6	0.0989	anti-DP4	0.0055
anti-DR9	0.2747	anti-DQ7	0.2088	anti-DP5	0.0110
anti-DR10	0.1538	anti-DQ8	0.3132	anti-DP13	0.0110
anti-DR11	0.1484	anti-DQ9	0.2967	anti-DP14	0.0165
anti-DR12	0.2802			anti-DP18	0.0055
anti-DR13	0.1923				
anti-DR14	0.1374				
anti-DR15	0.2033				
anti-DR16	0.2473				
anti-DR17	0.1868				
anti-DR18	0.1703				
anti-DR51	0.2912				
anti-DR52	0.1209				
anti-DR53	0.1868				
anti-DR103	0.1923				

## Discussion

Many studies have demonstrated the importance of HLA antigens in the transplantation of organs and the effect of anti-HLA antibodies on survival and graft rejection [1]–[3], [13]. For this reason, before transplantation, there is a need to ensure the best immunological compatibility between donor and recipient. In addition to compatibility, it is necessary to determine the existence of preformed antibodies, since the presence of these antibodies in the recipient against the donor’s specific antigen, can result in a deleterious response to transplantation [1], [9]. Therefore, the determination of the specificity of anti-HLA antibodies in the serum is of paramount importance for patients with renal disease and transplant candidates.

This study provides the first data on the immunological profile of anti-HLA class I (A, B and Cw) and class II (DR, DQ and DP) antibodies and the percentage of PRA in renal transplant candidates from southern Brazil. The production of anti-HLA antibodies occurs as a result of sensitizing events [4]–[8], and individuals may differ in the development of these antibodies. Unlike the results of studies conducted in other countries [14]–[16], most of the patients in this study were positive for PRA (67.7%). However, the methodologies used to determine the PRA differ among studies. The high number of patients sensitized to HLA antigens can also be explained, in part, by the practice, in Brazil, of performing blood transfusions in both hypersensitive and non-sensitized patients without regard to the high probability of generating anti-HLA antibodies.

Silva et al. [17] reported high rates of leukocyte alloimmunization (30.5%) in a study conducted with 393 patients awaiting kidney transplantation in northeastern Brazil. In the present study, the percentage of sensitized patients was higher than that found by Silva et al. [17]. One possible explanation could lie in the different methodologies used to determine the PRA. Silva et al. used a complement-dependent microlymphocytotoxicity test sensitized with human antiglobulin, and the sera were tested against panel-reactive antibodies (PRA) consisting of 50 individuals phenotyped with HLA class I antigens.

Human leukocyte antigen alloimmunization in candidates for renal transplantation is a reality [18], [19] and knowledge of the incidence of sensitization to HLA antigens in the Brazilian population may be important for advances in renal transplantation and to minimize the waiting time for candidates [19].

Several studies have reported that pregnancies, blood transfusions and previous transplants are potential risk factors for development of anti-HLA antibodies [6], [20]. The vast majority of patients investigated in the present study had received blood transfusions (59.5%), some patients had received previous transplants (7.4%), and most women had had pregnancies (79.4%).

Pregnancy as an immunogenic factor inducing the mother to produce antibodies against the father’s antigens, which are carried by the fetus, has been reported in several studies [4], [5]. Heise et al. (2001) [14], Ozdemir et al. (2004) [15], and Karahan et al. (2009) [16] found an association between pregnancy and the production of anti-HLA antibodies. In this study, the vast majority of female patients had been pregnant and had become sensitized in this process. However, in the present study, the PRA-positive and negative groups did not differ in this potential risk factor for the development of anti-HLA antibodies. A possible explanation could lie in the presence of maternal HLA compatibility with the father, or the degree of immunogenicity of HLA antigens that the mother may have contacted through the fetus.

Blood transfusion remains an important factor in patients on hemodialysis. Although the use of recombinant human erythropoietin in hemodialysis patients has resulted in improved hemoglobin levels and ameliorated the symptoms of anemia, significantly reducing the number of transfusions required, its use does not completely eliminate the need for blood transfusions in patients [21]–[23].

A few patients in the study had undergone previous transplants. Great progress has been made in organ transplantation, both in immunosuppressive therapy and in pretransplant laboratory tests. This progress is reflected in the reduced number of rejections, consequently reducing the number of patients who return to the waiting queue after an unsuccessful transplantation. Phelan et al. (2012) [24] followed 2381 kidney transplants, and observed that only 190 patients suffered a rejection. The same authors reported a decrease in cases of rejection over the years, from 7% in 1990 to less than 1% in 2009.

However, in the present study, the PRA-positive and negative groups did not differ in potential risk factors for the development of anti-HLA antibodies, in contrast to findings in studies conducted in other countries [14]–[16]. However, this study was conducted with a sample of Brazilian patients with chronic renal failure, also differing from other studies, because of the relatively larger number of patients with a positive PRA compared with those with a negative PRA. This study also used a different methodology for determination of PRA and anti-HLA antibodies.

Among the PRA-positive patients, a high proportion were positive for both classes (I and II) of anti-HLA antibodies. Consequently, as expected, the number of HLA loci to which the antibodies were reactive was also high in the group with a PRA between 51% and 100% (group 2), in comparison with the PRA group of 1% to 50% (group 1), for the two classes of anti-HLA antibodies.

In this study also, a significant proportion of patients with a positive PRA showed values between 51% and 100%, demonstrating a high degree of sensitization to HLA in both class I and class II. The present study also found hypersensitized patients (patients with PRA greater or equal to 80). Patients with high percentages of PRA may have a higher probability of rejection [10]–[12].

Our study also documented the frequency anti-HLA-Cw and anti-HLA-DP antibodies, which are generally little explored in studies of antibody profiles. The clinical significance of pre-transplant anti-HLA-Cw and anti-HLA-DP has not been widely investigated, although studies have now reported the effect of HLA-Cw and -DP on the survival of the renal graft. Patients with mismatched HLA-Cw showed an increase in the likelihood of acute rejection [25], [26], hyperacute rejection [27], acute antibody-mediated rejection [28], [29], and chronic rejection [30] in the presence of anti-HLA-Cw. Qiu et al. (2005) [31] detected a higher frequency of anti-HLA-DP in patients who rejected their grafts than in those with functioning grafts, finding anti-HLA-DP in 5.1% of 138 renal transplant recipients with functioning grafts, and 19.5% of 185 patients with rejected grafts. They called attention to the fact that 13% of patients who had rejected the graft had only anti-HLA-DP antibodies [31]. Several studies have reported acute rejection [28], [32], [33] and antibody-mediated chronic rejection [34] in renal transplant recipients, in the presence of anti-HLA-DP. These occurrences emphasize the importance of knowledge of the specificities of these antibodies, in order to improve graft survival.

The frequency of HLA allele groups in this study was similar to that in healthy subjects from southern Brazil [35]–[37]. The Brazilian population is one of the most heterogeneous in the world, consisting of various ethnic groups [38], and the frequencies of HLA alleles may vary according to the predominant ethnic group in a region [36], [39], [40]. The samples tested in this study were from the state of Paraná in southern Brazil. Southern Brazil was settled mainly by Europeans, but also has significant numbers of people of African descent and Native Americans in the population [39]. This influence can be observed in the results of this study, which revealed a predominant contribution of HLA allele groups of European origin, such as HLA-A*24 and -B*44 [41]–[43], as well as the occurrence of allele groups of African influence such as HLA-A*30 and -B*15 [44], [45].

The most common HLA allele groups were HLA-A*02, -A*24, -A*01, -B*44, -B*35, -B*15, -DRB1*11, -DRB1*04 and -DRB1*13. In contrast, the specificities of anti-HLA loci for A, B and DR were frequently A34, A24, A68, B57, B58, B27, DR51, DR12 and DR9. There were similarities but also differences among the HLA allele groups, and the specificities of anti-HLA antibodies found in this study. This pattern is in agreement with the results found in another study of correlations between the frequency of HLA antigens and anti-HLA antibodies in renal transplant candidates [46]. HLA mismatch generally produces an antibody specific to that antigen. One would expect that the frequency of a specificity of anti-HLA antibody would correlate naturally with the frequency of the phenotype in the population, or if a HLA phenotype is present in low frequency in the population, antibodies specific for this phenotype would also be rare. However, Idica et al., 2006 [47] showed that immunization against low-frequency phenotypes may occur frequently, in agreement with the results found here. In the present study, relatively rare antigens such as A34, A68, B58, DR9, DR12 were among those that most induced the production of anti-HLA antibodies. This finding can also be explained by the mixture of ethnic groups, leading to a variety of combinations of HLA alleles and haplotypes in the population, and increasing the likelihood of HLA incompatibilities and sensitization to HLA antigens.

Heise et al. (2001) [14] and Karahan et al. (2009) [16] showed that certain HLA phenotypes may be associated with the development of anti-HLA antibodies in patients with chronic renal failure. Karahan et al. (2009) [16] showed that the frequencies of the HLA-A3, HLA-A66 and HLA-B18 phenotypes were significantly elevated in PRA-positive patients compared to PRA-negative patients, and the DRB1*07 allele was more frequent in patients with a history of events and no production of anti-HLA antibodies, suggesting that DRB1*07 may be associated with a low risk of formation of anti-HLA antibodies. Heise et al. (2001) [14] found a positive association for the development of anti-HLA antibodies with HLA class I phenotypes (HLA-A10, -A19, -A36, -B42 and -B53), and negative associations with HLA class I phenotypes (A1, A2, A11, B8, B12, B40) and class II phenotypes (DR1, DR4, DR7). The same study found that the DR3 phenotype was associated with high values of PRA and lower rates of graft survival, and the DR1 and DR4 phenotypes were associated with low values of PRA and good graft survival. However, some phenotypes such as HLA-A2 and HLA-B18, which showed positive or negative associations in these studies, are common both in the chronic renal failure patients in this study and in populations of healthy individuals [36], [37]. These studies emphasize the importance of knowledge of HLA diversity and the profile of antibodies to the HLA system, in the context of organ and tissue transplantation.

The limitations of this study consist particularly in the use of small sample sizes, and in the difficulties in obtaining relevant information (pregnancies, blood transfusions and previous transplants), because the majority of hemodialysis clinics and transplant centers in Brazil do not have a system for efficient data storage, updating and retrieval of this information. Despite these limitations, this study provided important data on sensitization to HLA antigens, including pre-transplant anti-HLA-Cw and anti-HLA-DP sensitization, which is little explored in studies of anti-HLA antibody profiles.

## Conclusions

The data from this study document the profile of anti-HLA antibodies in chronic renal failure patients on the waiting list for an organ in northern and northwestern Paraná, showing a high proportion of sensitization to HLA antigens (positive PRA). Evaluation of the immune response to HLA antigens in the pre-transplant workup may contribute to future studies of the association of HLA alleles with the development of anti-HLA antibodies in Brazilian patients, patients with chronic kidney disease, and for the understanding of future events related to the acceptance or rejection of grafts.
